# Cerebral hemorrhage after left ventricular assist device implantation in a patient with dilated cardiomyopathy: a case report

**DOI:** 10.3389/fcvm.2026.1810251

**Published:** 2026-07-20

**Authors:** Yanwei Wei, Yunqiang Zhang, Li Han, Aixue Zhang, Rui Jing

**Affiliations:** 1 Department of Heart Failure, TEDA International Cardiovascular Hospital, Tianjin University, Tianjin, China; 2 Cardiac Care Unit, TEDA International Cardiovascular Hospital, Tianjin University, Tianjin, China

**Keywords:** advanced heart failure, blood pressure management, cerebral hemorrhage, dilated cardiomyopathy, left ventricular assist device, warfarin

## Abstract

**Background:**

For patients with end-stage heart failure who have undergone left ventricular assist device (LVAD) implantation, postoperative complications such as cerebral hemorrhage are common, leading to high mortality and complex treatment. Effective blood pressure management and adjustment of anticoagulation targets are crucial in the treatment of cerebral hemorrhage after LVAD implantation.

**Case presentation:**

We report the case of a 66-year-old male patient who suffered from a hemorrhagic stroke shortly after the implantation of a domestic LVAD. Intensive blood pressure control was initiated, and the anticoagulation regimen was precisely adjusted. These measures prevented the occurrence of poststroke sequelae, reduced the risk of death, and significantly improved the patient's quality of life. As of the current 5-year follow-up, the patient remains in good general condition.

**Conclusion:**

This case provides valuable insights for clinicians managing LVAD patients who experience stroke.

## Introduction

Left ventricular assist device (LVAD) implantation has gradually become an established treatment option for patients with end-stage heart failure (1). Neurological complications are among the most common postoperative complications following LVAD implantation, characterized by acute onset, high disability rate, and high mortality, necessitating urgent treatment. Rational blood pressure control and adjustment of anticoagulation therapy are critical. This report describes a case of hemorrhagic stroke (HS) complication that occurred early postoperatively (36 days postoperative, POD 36) in a patient implanted with a domestically produced LVAD. During the acute phase of cerebral hemorrhage, we actively implemented an intensive antihypertensive therapy regimen, achieving a target mean arterial pressure (MAP) of 60–65 mmHg. In addition, warfarin anticoagulation therapy was actively restarted on the third day after cerebral hemorrhage, with the international normalized ratio (INR) maintained within the lower limit of 2.0–2.2.The patient's cerebral hemorrhage was controlled without thrombotic events, with a favorable prognosis and stable cardiac function. Three years postoperatively (1,183 days), the patient experienced another ischemic stroke event, yet neither event resulted in severe sequelae. The patient remained in good general condition during a 5-year postoperative (POY 5) follow-up, with no further hospitalization due to worsening heart failure. This case report summarizes the patient's diagnosis, treatment course, and follow-up results, aiming to provide clinicians with practical experience in managing such complex scenarios.

## Case presentation

**Figure 1:**
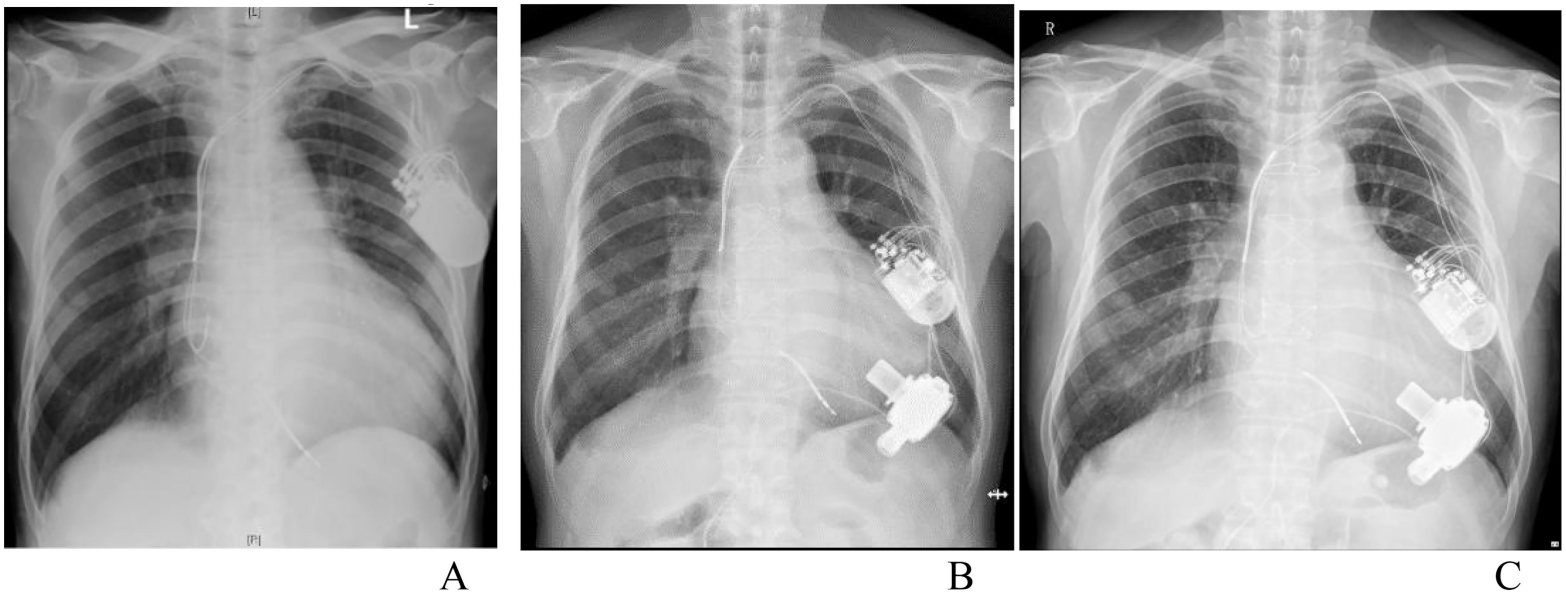
Changes in chest radiographs. **(A)** At the time of admission, the cardiac shadow was enlarged. After the implantation of a cardiac resynchronization therapy defibrillator, the pulmonary congestion was improved. **(B)** On 24 February 2023 (POY2.5), the cardiac shadow was reduced, and the pulmonary congestion was improved. **(C)** On 21 March 2025 (POY4.5), no significant change in the cardiac silhouette was observed.

**Figure 2:**
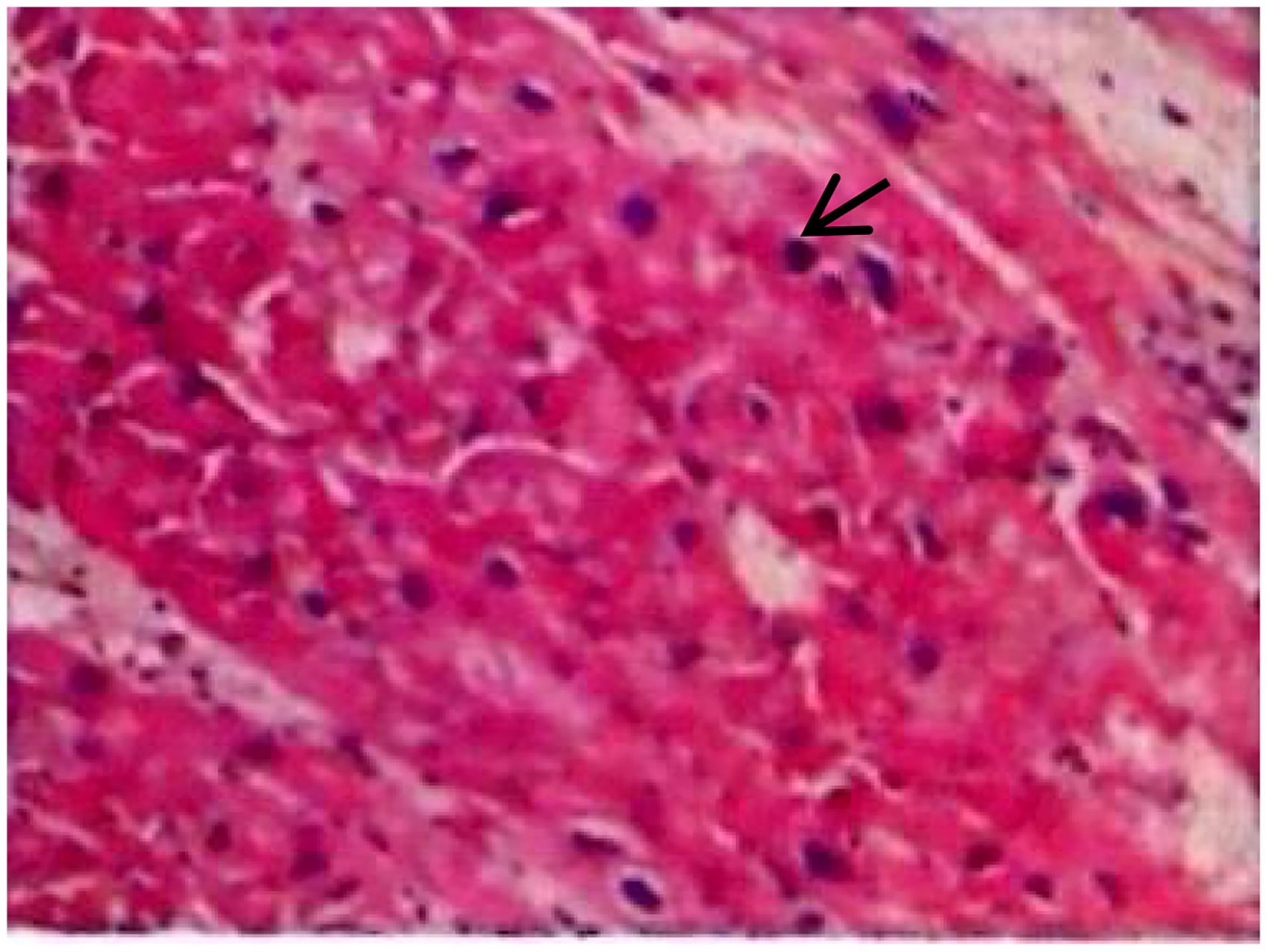
Myocardial biopsy findings during LVAD implantation. The size of the left ventricular apex was 1.6 cm × 0.8 cm × 0.2 cm, and the section was light brown and soft. Myocardial biopsy showed myocardial cell degeneration, hypertrophy, enlarged nuclei, hyperchromatism, and interstitial fibrous tissue hyperplasia, along with a small amount of chronic inflammatory cell infiltration (H&E staining, ×10).

A 66-year-old male patient was admitted to the hospital on 12 September 2020, with “intermittent chest tightness for 10 years, aggravated for 9 h.” He had experienced chest tightness and shortness of breath after activity for 10 years before admission, which could be relieved by rest. Three years ago, the local hospital diagnosed the patient with dilated cardiomyopathy (DCM) and heart failure and treated him with a cardiac resynchronization therapy defibrillator. Since 7 January 2020, the patient had been hospitalized more than 10 times due to increased dyspnea, decreased activity tolerance, weakness, anorexia, nausea, and lower limb edema after exercise. He had experienced shortness of breath and paroxysmal palpitations accompanied by dyspnea after the standardized antiheart failure drug treatment in another hospital. In addition, he experienced sudden dyspnea and orthopnea 9 h before admission. He had no history of hypertension, diabetes, or hyperlipidemia. He had a 40-year smoking and light drinking habit. His physical examination revealed the following: pulse 80 bpm, respiration 22/min, blood pressure 97/50 mmHg, body mass index 21.8 kg/m^2^, and body surface area 1.73 m^2^. Lying down with a high pillow, clear consciousness, jugular venous engorgement, positive hepatojugular venous reflux sign. The breath sounds of both lungs were coarse, with moist rales. The cardiac border was enlarged bilaterally, heart rhythm was regular, and a systolic ejection murmur could be heard at the mitral valve auscultation area. The abdomen was soft, without tenderness or rebound pain. The liver was enlarged and palpable 2 cm below the costal margin but not below the xiphoid process. The spleen was not palpable below the costal margin. Pitting edema was present in both lower extremities. Laboratory tests (reference range) revealed the following: N-terminal pro-B-type natriuretic peptide (NT-proBNP) 5,484 pg/mL (0–125 pg/mL), D-dimer 1.40 mg/L (0–0.50 mg/L), creatinine 125 μmol/L (30–106 μmol/L), total bilirubin 22.8 μmol/L (1.7–20 μmol/L), direct bilirubin 9.4 μmol/L (0–6.8 μmol/L), and indirect bilirubin 13.4 μmol/L (3.4–17.1 μmol/L). Other routine tests—blood, urine, stool, blood lipids, electrolytes, creatine kinase isoenzymes, creatine kinase, myoglobin, troponin, and thyroid function—showed no significant abnormalities. Echocardiography demonstrated that the left ventricular end-diastolic diameter was 90 mm, the left atrial anteroposterior diameter was 51 mm, the right atrial diameter was 35 mm, the left ventricular ejection fraction was 24%, the pulmonary artery systolic pressure was 73 mmHg, and the inferior vena cava diameter was 22 mm. The motion of the interventricular septum and the left ventricular wall was reduced to varying degrees. There was anterior leaflet prolapse of the mitral valve with moderate-to-large reflux, and tendon rupture was considered the cause. Left ventricular diastolic and systolic functions were reduced, with severe pulmonary hypertension. ECG revealed sinus rhythm with atrial-sensed ventricular pacing, complete left bundle branch block, and a QRS duration of 161 ms. Chest radiograph showed an enlarged heart shadow and pulmonary congestion (Figure 1A). Cranial CT revealed scattered lacunar foci bilaterally in the basal ganglia and centrum semiovale. Coronary CT angiography revealed no abnormalities and ultrasound examination of the cervical and lower limb vessels was also unremarkable. Given the patient’s DCM, end-stage heart failure, New York Heart Association (NYHA) cardiac function class IV, and the National Institutes of Health-Interagency Society for Mechanical Circulatory Support (INTERMACS) heart failure Class 2 status, along with a prognostic assessment indicating a 1-year mortality rate of 26.9% (Maggic), there were indications for LVAD implantation without contraindications. After improving cardiac function through drug therapy, LVAD implantation (rotational speed: 2,400 rpm, flow: 3.25 L/min, power: 3.62 W) was performed, along with mitral valvuloplasty and tricuspid valvuloplasty on 22 September 2020. Endocardial biopsy (left ventricular apex) taken intraoperatively showed degeneration, hypertrophy, large nuclei, hyperchromatic cardiomyocytes, hyperplasia of interstitial fibrous tissue, and a small amount of chronic inflammatory cell infiltration (Figure 2). The patient was treated with heparin as a bridge to warfarin therapy, along with aspirin for platelet aggregation and sacubitril–valsartan sodium for blood pressure reduction. The mean blood pressure (MAP) was controlled at 70–80 mmHg, and the INR was maintained between 2 and 2.5. The patient recovered smoothly 1 month after the operation.

**Figure 3:**
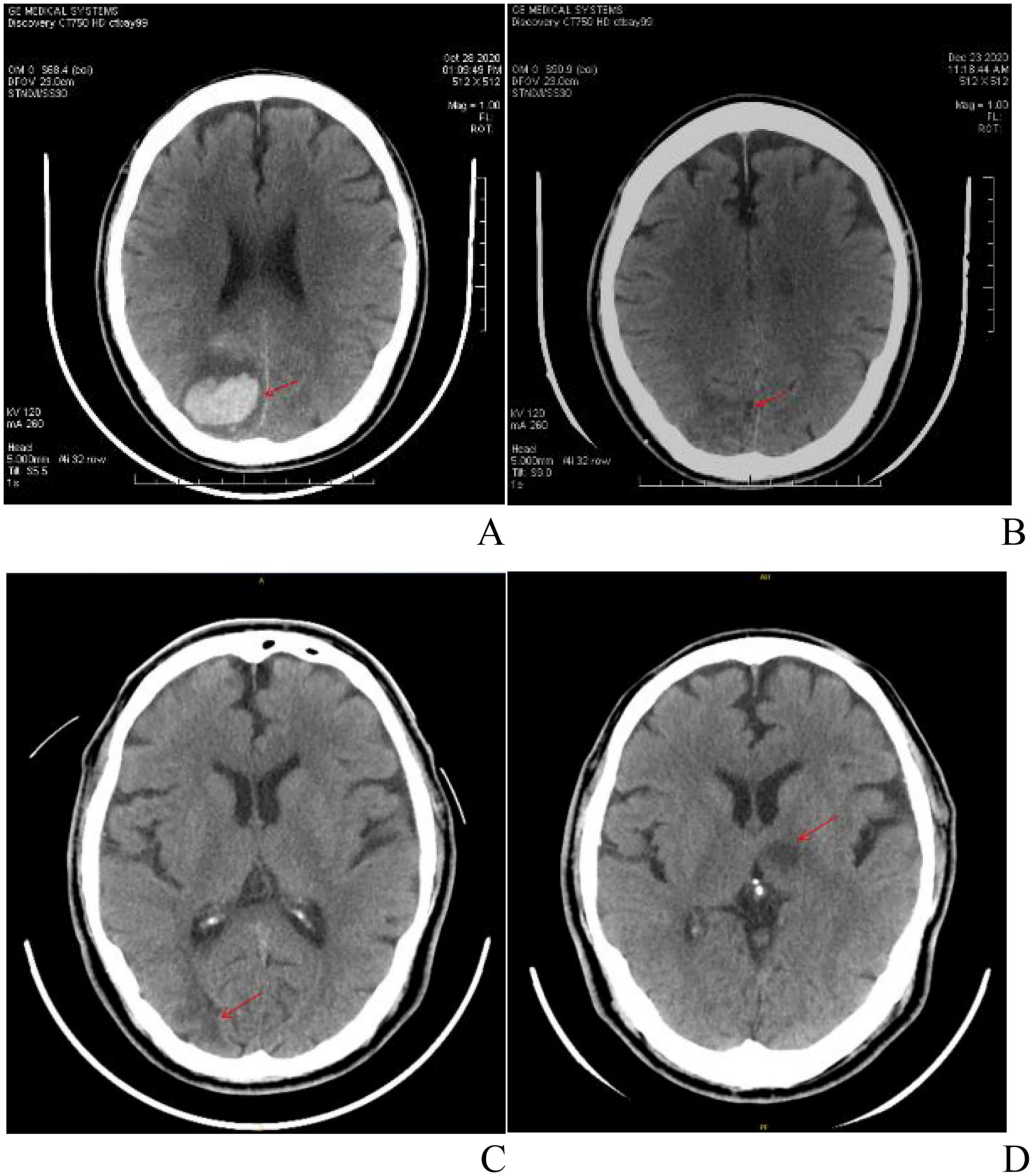
Brain CT of patient with stroke. **(A)** Right parieto-occipital lobe hemorrhage (volume: approximately 15 mL). **(B)** Right parieto-occipital lobe hemorrhage was absorbed, and a local soft focus formed. **(C)** Soft focus in the right parieto-occipital lobe, suspicious low-density shadow in the right semiovoid center. **(D)** Left thalamo-cerebral peduncle infarction, right parieto-occipital encephalomalacia, and right hemispheric center suspicious hypodensity roughly anterior (arrow).

However, on 28 October 2020, 36 days after LVAD implantation, at approximately 2:00 a.m., the patient experienced right-sided headache upon waking from sleep, accompanied by morning visual blurring, fatigue, and clear consciousness but poor mental status. Blood pressure measured at 6:00 a.m. was 96/78 (84) mmHg, pulse rate was 84 beats/min, body temperature was 36.0°C, and respiratory rate was 19 breaths/min. Examination showed the absence of temporal and nasal fields in the left and right eyes. The NIH Stroke Scale (NIHSS) score was 2 and the modified Rankin Scale (mRS) score was 1. Urgent examination showed hemorrhage in the right parieto-occipital lobe (about 15 mL) (Figure 3A). The patient was diagnosed with acute cerebral hemorrhage in the right parietal-occipital lobe. Warfarin and Aspirin were immediately stopped, and intensive antihypertensive treatment was administered. MAP was strictly controlled between 65 and 70 mmHg to ensure adequate systemic perfusion, while avoiding postural hypotension, oliguria, and cool extremities. On the morning of 29 October 2020, the patient experienced drowsiness, accompanied by persistent headache, fatigue, and malaise. The hemianopia symptoms improved, and the 45-degree visual fields in the temporal region of the left eye and the nasal region of the right eye recovered compared to previous levels. On 30 October 2020, follow-up cranial CT revealed that there were increased mass-like and linear hyperdense lesions in the right parieto-occipital lobe. The hemorrhage volume was estimated at approximately 22 mL, and the peripheral patchy edema zone had expanded. Dehydration and intracranial pressure reduction therapy were continued. On 31 October 2020, cranial CT showed a slight reduction in mass-like and linear hyperdense lesions in the right parieto-occipital lobe. The hemorrhage volume decreased to about 20 mL, and the edema zone was slightly reduced. Following LVAD implantation, this patient exhibited high risks of pump thrombosis and embolism. After a multidisciplinary discussion at the hospital, 1 mg of oral warfarin was administered as anticoagulant therapy at 00:28 on 1 November 2020 (the third day posthemorrhage), aiming to maintain the MAP at 65–70 mmHg and the INR at 2.0–2.2.The patient exhibited good systemic perfusion, improved mental status, and a gradual alleviation of fatigue and discomfort. At 15:00 on the same day, INR was rechecked and found to be 1.61. To prevent further bleeding, the target MAP was adjusted to 60–65 mmHg. At 22:00, 1 mg of warfarin was orally administered again, accompanied by an intravenous infusion of heparin sodium for bridging therapy. The activated partial thromboplastin time was monitored, with a target range of 45–50 s. On 2 November 2020, the patient presented with headache, fatigue, drowsiness, and discomfort as previously reported. The left visual field showed improvement compared to previous assessments. Cranial CT revealed no significant changes in the mass-like and linear high-density lesions in the right parieto-occipital lobe, with a hematoma volume of approximately 20 mL. The volume of hemorrhage and edema did not expand further. The mean arterial pressure target was adjusted upward to 65–75 mmHg. On 3 November 2020, the patient's symptoms improved, with an expanded left visual field compared to previous measurements. On 9 November 2020, the patient no longer experienced fatigue, headache, or drowsiness, and there were no significant changes in the left visual field. Follow-up cranial CT revealed a reduced mass-like hyperdense lesion in the right parieto-occipital lobe. The lesion had blurred margins, a hemorrhage volume of approximately 9 mL, and a smaller edema zone compared to previous findings. A follow-up cranial CT scan on 23 December 2020, demonstrated that the right parieto-occipital lobe hemorrhage had been largely absorbed, with the formation of localized softening foci (Figure 3B). The NIHSS score was 1 and the mRS score was 1. One month after LVAD implantation, the patient experienced fever, with a positron emission tomography–computed tomography (PET-CT) examination indicating mediastinal infection. Subsequently, exploration of the pericardium and mediastinum with placement of a drainage tube was performed. Postoperative management included anti-infective therapy, mediastinal lavage and drainage, continuous negative pressure suction at the thoracic incision site, and regular wound dressing changes, which resulted in clinical improvement. In addition, minor nasal mucosal bleeding occurred sporadically in the early postoperative period, with minimal blood loss that could be controlled by local compression. The patient was discharged on 17 December 2020, and continued regular administration of warfarin, spironolactone, sacubitril–valsartan, and metoprolol. Remote electrocardiographic monitoring was used outside the hospital to monitor vital signs and LVAD mechanical parameters daily, ensuring stable blood pressure, anticoagulation, and device functioning. No recurrent infections, hemorrhage, right heart failure, pump thrombosis, or arrhythmias occurred. The patient demonstrated good tolerance to LVAD, with stable vital signs and no further hospitalization due to worsening heart failure. His quality of life improved significantly.

**Figure 4:**
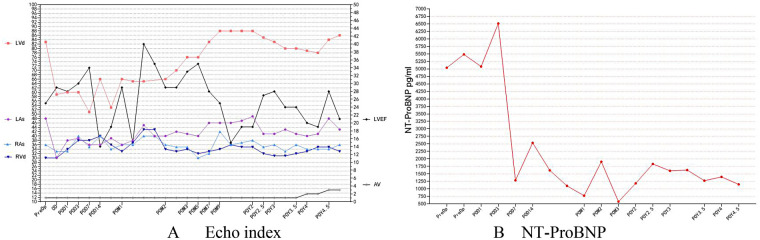
Changes of echocardiographic parameters and NT-ProBNP in patients. **(A)** Echo index. **(B)** NT-ProBNP. AV, aortic valve opening; 1, AV not opening; 2, AV sometimes opens; 3, AV opens slightly with each beat.

The patient was followed up for 3 months postoperatively (POM 3). Echocardiography demonstrated a left atrial end-systolic diameter of 41 mm, a left ventricular end-diastolic diameter of 76 mm, and a left ventricular ejection fraction of 33%. The NYHA cardiac function was Class I, and physical tolerance was good. At the 6-month follow-up, the patient's visual field had completely recovered, and neurological examination showed no abnormality, with an NIHSS score of 0 and an mRS score of 0. Follow-up was conducted every 6 months, with no discomfort reported by the patient. The right atrium and ventricle were normal in size, and the right heart function was normal as well. The left atrial end-systolic diameter fluctuated between 40 and 49 mm. The left ventricular end-diastolic diameter fluctuated between 78 and 88 mm, and the left ventricular ejection fraction fluctuated between 15% and 28% (Figure 4A). NT-proBNP levels were maintained at low levels (Figure 4B), and NYHA functional class remained stable at Grade I. On 24 February 2023 (2.5 years postoperatively, POY 2.5), follow-up chest X-ray revealed a reduced cardiac silhouette and improved pulmonary congestion compared to previous findings (Figure 1B). At the final follow-up on 21 March 2025 (POY 4.5), no significant changes in cardiac silhouette were observed compared to previous findings (Figure 1C). The MAP was controlled at 65–70 mmHg, and the INR was controlled at 2.0–2.5. The patient's condition was stable, but he had persistent left ventricular dilatation, with no significant improvement in cardiac function. Although the application of LVAD could alleviate the patient's symptoms and significantly improve his quality of life, it could not achieve myocardial reverse remodeling, and cardiac function recovery was limited. Cardiac transplantation therapy was recommended. However, due to advanced age and a history of cerebral hemorrhage, surgical risks and costs were deemed high. After deliberation, the patient and his family members declined transplantation and chose to continue LVAD therapy.

On 19 December 2023, the patient was admitted to the hospital with “sudden speech impairment for 3 h.” The patient experienced a sudden visual blackout while sitting upright 3 h prior to admission and subsequently leaned forward onto the table. Although conscious, the patient was unresponsive to calls. Speech was restored after half an hour, though with dysarthria. Physical examination at admission revealed the following: T: 35.7℃, P: 70 bpm, R: 19 times/min, and BP: 87/70 mmHg. Neurological examination revealed that the pupils were 3 mm bilaterally with normal light reflexes. The tongue deviated to the right upon extension, and the jugular vein was normal. Hepatic and jugular venous reflux signs were negative. Breath sounds were clear in both lungs, with no moist rales and no pleural friction. The cardiac boundary was enlarged, the heart rhythm was regular, and the sound of blood pumping could be heard, steady without any additional noise. The radial pulse could not be felt. The abdomen was soft with abdominal tenderness but no rebound pain or muscle tension. The liver and spleen were not palpable under the ribs. Bowel sounds were normal, and there was no edema in either lower limb. Right-sided muscle strength was weakened (grade IV) but left-sided muscle strength was normal, and the bilateral pathological signs were negative. The NIHSS score was 4 and the mRS score was 2. Auxiliary examination demonstrated no significant abnormalities in routine blood, liver and kidney function tests. INR was 2.13, plasma free hemoglobin was 18 mg/L. Cranial CT (Figure 3C) revealed right parieto-occipital global malacia, suspected Hypodense Shadow in right semiovoid center, and ill-defined hypodense foci. Acute cerebral infarction was also considered. Edaravone was administered for neuroprotective therapy, while sacubitril/valsartan (Entresto) and benidipine were given to control blood pressure, with the MAP maintained at 65–70 mmHg. In addition, metoprolol tartrate, spironolactone, and empagliflozin were administered to improve heart failure management. After the aforementioned treatments, the patient's speech gradually returned to normal, and the right deviation of the tongue was significantly improved compared with the initial presentation. On 25 December 2023, cranial CT was re-examined (Figure 3D), suggesting left thalamic–cerebral peduncular infarction. The patient remained stable and was discharged with medication. He was told to discontinue diuretics and potassium supplements and continue sacubitril–valsartan, metoprolol tartrate tablets, spironolactone, empagliflozin, digoxin, rosuvastatin, and warfarin. Mean arterial pressure was maintained at 65–75 mmHg and INR at 2.0–2.5. On 1 November 2024, the patient underwent LVAD lead replacement surgery under general anesthesia due to aging of the device’s electrode leads. Postoperative recovery was smooth, and the patient’s overall condition was good. At the latest follow-up, 5 years after LVAD surgery, the patient's neurological examination was normal (NIHSS score 0, mRS score 0). He achieved long-term survival with LVAD, experienced no readmissions for worsening heart failure, and maintained stable cardiac function.

## Discussion

LVAD implantation has become an effective surgical treatment for patients with terminal heart failure, with 5-year survival rates not significantly different from those of heart transplantation (2). The LVAD field in China developed relatively late. The LVAD used in this case, the HeartCon, is a latest-generation, magnetically levitated centrifugal pump jointly developed by our institution and the China Academy of Launch Vehicle Technology. It was commercially released on 13 July 2022, and as of 1 January 2026, 380 implantations had been performed. Stroke remains the most severe complication associated with LVAD therapy and is a leading cause of postoperative death (3). Strokes can be categorized as either ischemic stroke (IS) or HS. Of the two, IS is more common, but HS has a higher hospitalization mortality rate (88.8% vs. 21.1%) (4) and a worse prognosis. According to the INTERMACS 2023 annual report from the Society of Thoracic Surgeons, between 2018 and 2022, a total of 900 (8.24%) neurological complications occurred among 10,920 patients implanted with all-magnetic suspension LVAD. The 1-year incidence rate was 6.7%, and the 2-year incidence rate was 9% (5). The MOMENTUM3 study demonstrated that the incidence of stroke, hemorrhage, and thrombosis in the new-generation centrifugal LVAD was lower compared with the axial-pump LVAD (6). However, the incidence of IS or HS remained as high as 8.6%–10.7% 1 year after implantation (7). Data from a single-center study using domestically produced LVAD reported (8) an incidence of neurological complications of 0.08 per person per year, which is close to the international average. The incidence of HS was 0.02 per person per year, significantly lower than the international average (0.03–0.04 per person per year). However, all five HS patients died during long-term follow-up. Among the 15 IS patients, 12 had no aftereffects after drug treatment, one had residual limbic disability, and two were left with speech impediment. The incidence of neurological complications following LVAD implantation at this center is comparable to international standards, with IS being the most common complication. However, domestic research on neurological complications after LVAD implantation remains relatively scarce.

There is currently no unified standard for target blood pressure control in patients with HS after LVAD implantation. International guidelines (9) recommend maintaining MAP below 80 mmHg in LVAD patients. In the acute phase of HS, high blood pressure is often present, closely associated with adverse outcomes such as enlarged hematoma, aggravated cerebral edema, and death. Early intensive blood pressure reduction is beneficial in preventing further expansion of the hemorrhage volume. The risk of recurrence of HS is high. The recurrence rate was 14.6% in a cohort study by Santos et al. (10) and 23.5% in a study by Cho et al. (11). However, blood pressure reduction treatment can significantly reduce the risk of HS recurrence. The hemodynamics of patients with LVAD implantation change, and they often have non-pulsatile flow. Therefore, treatment differs from the post-HS blood pressure management protocols used in patients without LVADs. In our case, the patient's MAP was initially controlled between 70 and 80 mmHg post-LVAD. Following the HS, we implemented intensive blood pressure reduction protocols. In the early stages, the MAP was strictly maintained between 60 and 65 mmHg. While ensuring blood supply to important organs, this approach effectively reduced the volume of hemorrhage and limited the expansion range of the hematoma. After HS absorption in the patient, the long-term MAP control target was adjusted to 65–70 mmHg. After precise and enhanced hypotensive therapy, HS in the patient was controlled, the prognosis was good, and no neurological sequelae remained.

Anticoagulant therapy is the cornerstone of postimplantation treatment for LVAD patients. The combination of aspirin and warfarin constitutes the most commonly used antithrombotic regimen following LVAD implantation (12). In the postoperative analysis of the ENDURANCE trial, both INR >3.0 and INR <2.0 were associated with an increased risk of stroke (13). Current international strategies for preventing hemorrhagic complications post-LVAD primarily focus on (1) reducing the aspirin dose or suspending aspirin and (2) reducing INR target values. The heartWare ventricular assist device (HVAD) study found that reducing the aspirin dose may increase the risk of pump thrombosis and stroke (14). However, the MOMENTUM 3 study showed that reducing the dose in HeartMate 3 implantation patients did not increase the risk of hemocompatibility events (15). The TRACE study initially validated the feasibility of low-intensity anticoagulation regimens in HeartMate 2 implantation patients (16). The ARIES study showed that omitting aspirin after surgery did not increase the risk of thrombosis and significantly reduced hemorrhagic events (17). Statistical data from our center showed that among patients supported by the domestically manufactured HeartCon LVAD, patients receiving the warfarin-only anticoagulation strategy achieved an average duration of sustained support of 458 days. The 1- and 2-year survival rates of patients after surgery were 95%, higher than the data published by INTERMACS (82.3% and 73.1%, respectively). Stroke incidence rate within 1 year (13.6%) was equivalent to the international average (12.7%) (18, 19). After LVAD implantation, patients need to take anticoagulant drugs for a long period of time. However, these drugs should be discontinued immediately after HS complications appear. Therefore, there is a clear conflict in the treatment aspect. In our case, the patient also had risk factors such as old age. Moreover, there are no relevant guidelines and expert consensus on clinical treatment to provide guidance. Reports are inconsistent about the recovery of anticoagulant treatment protocols after HS. Some studies have pointed out that the average time for anticoagulation recovery is 6.8–10.5 days without waiting (20). In contrast, other studies have proposed that the time for anticoagulation recovery after HS ranges from 6 to 14 days (10). Our center strictly controlled the patient's MAP at 60–65 mmHg and actively restarted warfarin anticoagulation treatment on the third day after surgery. The INR target range was set with a lower limit of 2.0–2.2. Through multidisciplinary collaboration, the patient gradually recovered without any remaining sequelae. The anticoagulation target in this case differed from international guidelines (21), which recommend maintaining an INR of 2.0–3.0. For this HS patient, the early INR control target was set at 2.0–2.2, and INR was maintained long-term at 2.0–2.5 after discharge.

The patient was regularly followed up and remained in generally good condition. However, there was no improvement in cardiac function, and myocardial reverse remodeling could not be achieved, resulting in pump dependence. Four years after the surgery, the LVAD device electrode wires were replaced due to aging. The patient refused heart transplantation and continues to live long-term with pump support. This article presents the detailed diagnostic and therapeutic course of this patient, discusses the management of neurological complications after LVAD implantation and outlines strategies for blood pressure and anticoagulation management. It aims to provide a reference for the treatment of similar patients in clinical practice.

## Conclusion

This case demonstrates that in patients with LVAD implantation who have experienced a stroke, particularly a hemorrhagic stroke, it is crucial to appropriately control blood pressure levels and adjust anticoagulation therapy.

## Data Availability

The datasets presented in this study can be found in online repositories. The names of the repository/repositories and accession number(s) can be found in the article/Supplementary Material.
